# State-of-the-art polymeric nanoparticles as promising therapeutic tools against human bacterial infections

**DOI:** 10.1186/s12951-020-00714-2

**Published:** 2020-10-31

**Authors:** Amanda Cano, Miren Ettcheto, Marta Espina, Ana López-Machado, Yolanda Cajal, Francesc Rabanal, Elena Sánchez-López, Antonio Camins, Maria Luisa García, Eliana B. Souto

**Affiliations:** 1 Department of Pharmacy, Pharmaceutical Technology and Physical Chemistry, Faculty of Pharmacy and Food Sciences, University of Barcelona, Av Joan XXIII, 27-31, 08017 Barcelona, Spain; 2 Institute of Nanoscience and Nanotechnology (IN2UB), Barcelona, Spain; 3 Biomedical Research Networking Centre in Neurodegenerative Diseases (CIBERNED), Madrid, Spain; 4 Department of Pharmacology, Toxicology and Therapeutic Chemistry, Faculty of Pharmacy and Food Sciences, University of Barcelona, Barcelona, Spain; 5 Unit of Biochemistry and Pharmacology, Faculty of Medicine and Health Sciences, University of Rovira I Virgili, Reus (Tarragona), Spain; 6 Section of Organic Chemistry, Department of Inorganic and Organic Chemistry, Faculty of Chemistry, University of Barcelona, Barcelona, Spain; 7 Department of Pharmaceutical Technology, Faculty of Pharmacy, University of Coimbra, Coimbra, Portugal; 8 CEB - Centre of Biological Engineering, University of Minho, Campus de Gualtar, 4710-057 Braga, Portugal

**Keywords:** Bacterial infections, Infectious diseases, Polymeric nanoparticles, Nanomedicine, Nanotechnology

## Abstract

Infectious diseases kill over 17 million people a year, among which bacterial infections stand out. From all the bacterial infections, tuberculosis, diarrhoea, meningitis, pneumonia, sexual transmission diseases and nosocomial infections are the most severe bacterial infections, which affect millions of people worldwide. Moreover, the indiscriminate use of antibiotic drugs in the last decades has triggered an increasing multiple resistance towards these drugs, which represent a serious global socioeconomic and public health risk. It is estimated that 33,000 and 35,000 people die yearly in Europe and the United States, respectively, as a direct result of antimicrobial resistance. For all these reasons, there is an emerging need to find novel alternatives to overcome these issues and reduced the morbidity and mortality associated to bacterial infectious diseases. In that sense, nanotechnological approaches, especially smart polymeric nanoparticles, has wrought a revolution in this field, providing an innovative therapeutic alternative able to improve the limitations encountered in available treatments and capable to be effective by theirselves. In this review, we examine the current status of most dangerous human infections, together with an in-depth discussion of the role of nanomedicine to overcome the current disadvantages, and specifically the most recent and innovative studies involving polymeric nanoparticles against most common bacterial infections of the human body.

## Highlights

- Infectious diseases kill over 17 million people a year, and pose a huge health and socioeconomic burden worldwide.
- Tuberculosis, diarrhoea, meningitis, pneumonia, sexual transmission diseases and nosocomial infections are the most severe bacterial infections, which affect millions of people worldwide.
- Antibiotic multiple resistance is exponentially increasing due to the indiscriminate use of antibiotic drugs.
- It is estimated that 33,000 and 35,000 people die yearly in Europe and the United States, respectively, as a direct result of antimicrobial resistance.
- Nanotechnological strategies have emerged in the recent years as a promising alternative to solve these problems and improve available antibiotics effectiveness.
- It is well reported that polymers possess by themselves antibiotic effect.
- State-of-the-art polymeric nanoparticles possess optimal physicochemical characteristics to become a therapeutic revolution against bacterial infections.

## Introduction

Infectious diseases kill over 17 million people a year over the world [1]. Bacterial infections have a large impact on public health. Drug-resistant bacteria, viruses, parasites and fungi cause 700,000 deaths every year worldwide [2]. The latest *Global Health Study* undertaken by the World Health Organization (WHO) dated from 2016, estimated that infectious and parasitic diseases represent 9.7% from the whole deaths worldwide. In this ranking, tuberculosis has been placed as the first cause of death among bacterial infections (2.3% of global deaths), followed by diarrhoeal bacterial diseases (2%), meningitis (0.5%), bacterial sexual transmission disease (syphilis, chlamydia and gonorrhoea, 0.2%) and encephalitis (0.2%) [1]. Similarly, the latest reports of the *Global Burden of Diseases* consortium highlighted that, in 2016, infectious diarrhoea was the eighth leading cause of death among all ages and the fifth leading cause of death among children, being *Shigella* and enterotoxigenic *Escherichia coli* the most frequent and mortal bacteria [3, 4]; incident cases of meningitis globally increased 2.82 million in 2016, being the *Pneumococcus* the largest cause of years of life lived with disability (YLDs) [5]; the number of tuberculosis deaths was 1.21 million among HIV-negative individuals and 0.24 million among HIV-positive individuals [6]. Furthermore, in 2017, 11.0 million sepsis-related deaths were reported worldwide [7].

Not surprisingly, these data are significantly higher than the global computation in developing countries, since the lack of universal health systems, public health problems and access to potable drinking water, together with the limited financial resources, aggravate the situation of these patients [1, 4]. On the other hand, the indiscriminate use of antibiotic drugs in recent decades worldwide has generated a global public health problem due to the emergence of multiple resistance against antibiotics by a high number of microorganisms [8]. It is predicted that, by 2050, multidrug resistant (MDR) bacteria will cause up to 10 million deaths annually, with a burden cost in the global economy reaching US$100 trillion [2].

**Fig. 1:**
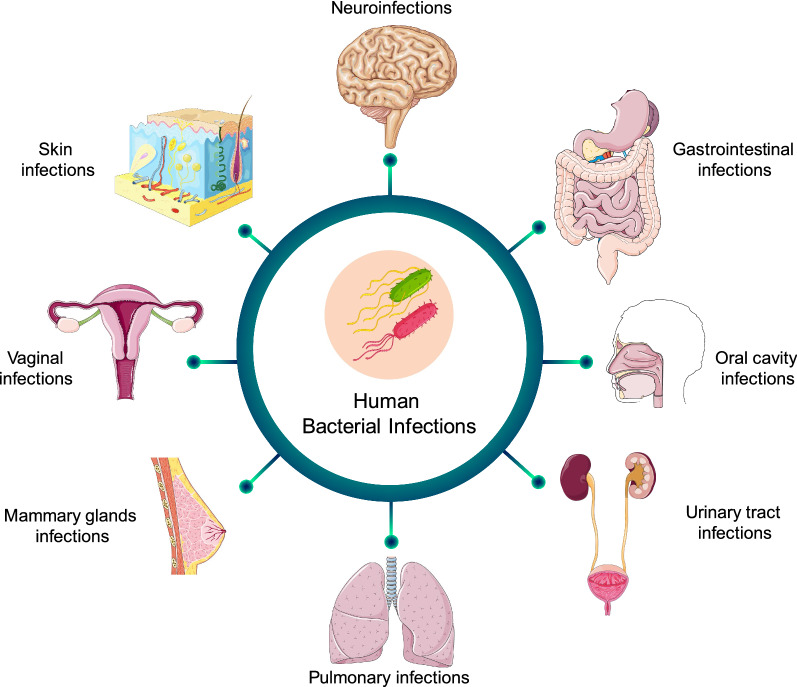
Most common human bacterial infections

Although the human body lives together with its own microbiota in an advantageous symbiosis, there are many bacteria pathogens capable to infect and colonize the human body and cause serious diseases (Table 1). Bacteria can be transmitted to humans through food, water, air, or living vectors. All of the human organs are susceptible to be infected by bacteria, but each species has a predilection for certain organs, and some organs are more sensitive to suffer bacterial infection (Fig. 1) [9]. "ESKAPE" bacteria is one of the groups of bacteria that are of greatest concern in the health environment due to several reasons. ESKAPE is an acronym encompassing the names of six most dangerous bacterial human pathogens (*Enterococcus faecium*, *Staphylococcus aureus*, *Klebsiella pneumoniae*, *Acinetobacter baumannii*, *Pseudomonas aeruginosa*, and *Enterobacter* species) commonly associated with a high antimicrobial resistance, which are responsible of the majority of nosocomial infections. Curiously, this acronym also references to the ability of these microorganism to escape the effects of commonly used antibiotics through evolutionarily developed mechanisms [10].

**Table 1:** Characteristics of most dangerous human infectious bacteria

Bacteria specie	Phylum	Order	Gram ±	Medium	Disease	Treatment	Morphology
*Enterococcus faecium*	Firmicutes	Lactobacillales	+	Aerobic/Anaerobic	Meningitis Endocarditis Nosocomial inf	Linezolid Daptomycin Tigecycline Streptogramins Sultamicillin	
*Staphylococcus aureus*	Firmicutes	Bacillales	+	Aerobic/Anaerobic	Skin inf Respiratory inf Nosocomial inf	Penicillin Oxacillin Flucloxacillin Kanamycin Gentamicin Streptomycin	
*Pseudomonas aeruginosa*	Proteobacteria	Pseudomonadales	−	Aerobic/Anaerobic*	Sepsis syndromes Pneumonia Nosocomial inf	Aminoglycosides Quinolones Cephalosporins Carboxypenicillins Ureidopenicillins Carbapenems Polymyxins Monobactams	
*Klebsiella pneumoniae*	Proteobacteria	Enterobacterales	−	Anaerobic^¥^	Nosocomial inf	Aminoglycosides Cephalosporins	
*Acinetobacter baumannii*	Proteobacteria	Pseudomonadales	−	Aerobic	Nosocomial inf Meningitis	Imipenem Meropenem Polymyxins	
*Enterobacter spp.*	Proteobacteria	Enterobacterales	−	Anaerobic	Nosocomial inf Urinary inf Respiratory inf	Cefepime Imipenem Aminoglycosides Quinolones Polymyxins	
*Mycobacterium tuberculosis*	Actinobacteria	Actinomycetales	±	Aerobic	Tuberculosis	Isoniazid Rifampin Pyrazinamide ethambutol Bedaquiline	
*Clostridium difficile*	Firmicutes	Clostridiales	+	Aerobic	Diarrhea Intestinal Inflammation	Stop antibiotic therapy Vancomycin Metronidazole Fidaxomicin	
*Chlamydia trachomatis*	Chlamydiae	Chlamydiales	−	Aerobic	trachoma lymphogranuloma venereum nongonococcal urethritis cervicitis salpingitis pelvic inflammatory disease blindness	Tetracycline Doxycycline Azithromycin erythromycin Ofloxacin	
*Neisseria meningitidis*	Proteobacteria	Neisseriales	−	Aerobic	Meningitis	Cephalosporins Penicillin G Chloramphenicol Corticosteroids	
*Vibrio cholerae*	Proteobacteria	Vibrionaceae	−	Anaerobic^¥^	Cholera	Hydration, glucose and electrolytes Tetracycline chloramphenicol	

*Commonly aerobic/less common facultative anaerobic

^¥^Facultative anaerobic

Other of the most dangerous bacteria for humans are *Neisseria meningitides* and *Vibrio cholera,* both belonging to the proteobacteria phylum *N. meningitides* is an exclusively human pathogen, causing bacterial meningitis in children and young adults, which finally lead to the death of the ten percent of the cases [11]. People with confirmed *N. meningitidis* infection should be hospitalized immediately for antibiotic treatment due to the severity of the symptoms [12]. On its behalf, *V. cholerae* causes cholera in humans. This pathogen is usually transmitted through the ingestion of contaminated food or water [13]. *V. cholerae* infection is limited to the intestinal tract, where disease symptoms are primarily caused by its toxins. It is mostly found in developing countries, since the lack of access to drinking water promotes its expansion, becoming an endemic disease in these regions [14].

*Clostridium difficile* is the most important cause of pseudomembranous colitis, an infection of the colon, often secondary to the eradication of the saprophyte microbiota. Antibiotics, especially broad-spectrum antibiotics, cause an imbalance of the intestinal flora, leading to an overpopulation of *C. difficile*, which in turn promotes the pseudomembranous colitis [15]. Due to the severity of this infection, monoclonal antibodies and drugs such Surotomycin, Rifamixin or Cadazolid are being studied in clinical trials as promising alternatives to currently available treatments [16].

*Mycobacterium tuberculosis* is responsible for the largest number of tuberculosis cases and the first cause of death in the world due to bacterial infection. Humans are the only known reservoirs of *M. tuberculosis*, which causes severe infection in lungs, but also spreading to other organs [17]. Importantly, the unusual structure and chemical composition of the mycobacterial cell wall deeply difficult the efficacy of the treatments, since drug penetration is almost impossible. [18].

Finally, *Chlamydia trachomatis* is an obligate intracellular bacterium that can replicate only within a host human cell [19]. *C. trachomatis* causes trachoma, oculogenital infections, nongonococcal urethritis and pneumonia. Moreover, *C. trachomatis* is the most common infectious cause of blindness and the most common sexually transmitted bacterium [20].

All these facts together highlight the global importance and severity of the antibiotic resistance issue, which arises the need to find new alternatives for these patients. In this sense, nanomedicine has become in the last decades in a revolutionary therapeutic tool [21]. The ability of controlled drug delivery systems to carry inside different antibiotics, enhance their solubility, target them to a specific organ and improve its penetration has opened a window of opportunities to overcome the disadvantages of the current antibiotic treatments and novel potential molecules [21]. Moreover, the possibility of accumulate the drug at the site of infection for a longer time, as well as avoiding the escape mechanisms of bacteria generate a great interest among the scientific community [21].

Specifically, polymeric nanoparticles (NPs) have emerged as a promising alternative mainly due to the polymeric structure. Polymeric materials have exhibited to possess antibiotic effects [22]. Among their several effects, it is highlighted their ability to accumulate onto the cell membranes, which finally promote the destruction of the entire bacteria cell. These polymer effects together with antibacterial properties of loaded drugs confers to these nanosystems many advantages as a multitarget approach against different infections [22]. Thus, in this review we deeply investigate and describe the main advantages of nanomedicine, specifically the state-of-the-art polymeric NPs, against most common human bacterial infections, as well as recent advances in novel drugs-loaded polymeric NPs as antibiotic therapies.

## Nanoparticles to improve antibacterial therapies

In the 1940s, penicinils promoted a revolution in the therapy of the twentieth century, significantly contributing to the control of infectious diseases, which were the leading cause of human mortality so far [23]. Due to this therapeutic success, over the last 50 decades, there was an indiscriminate use of these drugs, which led to several antibiotic resistances by many bacterial species, which implies a serious public health risk. Spontaneous modifications in the genetic material of these microorganisms improved their adaptation to the environment and the development of molecular mechanisms that limit the action of these agents, such as the production of degrading enzymes or the increase in their efflux pumps [10]. It is estimated that 33,000 and 35,000 people die yearly in Europe and the United States, respectively, as a direct result of antimicrobial resistance [24, 25].

For these reasons, the is a current need to improve the biopharmaceutical properties of existing compounds and also to find new, more effective antibacterial agents. Recent advances in nanotechnology, particularly the design of novel nanoparticles (NPs) as controlled drug delivery systems, have supposed a promising alternative impact in the general medicine, and specifically in the antimicrobial therapy [26]. NPs, controlled drug delivery systems with a diameter of 1–1000 nm and polymeric core, may offer remarkable improvements in the biopharmaceutical properties of antibacterial agents. Notably, they can provide an enhancement of drug solubility, modulation of drug release and delivery, promotion of stealth for immune evasion, targeting of antibacterial molecules to desired organs and tissues and increase the retention time in-situ, as well as the simultaneous delivery of multiple drugs [27]. Such unique advantages allow these systems to improve the therapeutic index of drug payloads when compared with free drug counterparts, perform an efficient focused therapy at the site of infection, increasing the penetration of loaded molecules through the bacteria cell wall, thus opening a window against antibacterial resistance [28].

**Fig. 2:**
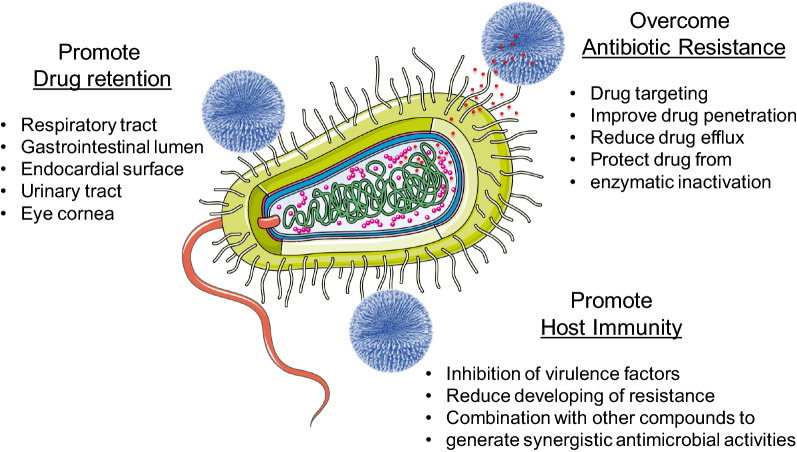
Main improvements brought by nanoparticles for the antibacterial therapy

Different strategies by which NPs can improve antibacterial therapy efficacy have already been described (Fig. 2) [29, 30]. The most important strategy is the antibiotic localization to the pathogen cell. This allows to reduce the therapeutic doses of the drugs, maintaining their efficacy and reducing the adverse effects [26]. The modulation of drug-pathogen interaction to overcome antibiotic resistance is another of the most effective strategies. For example, liposomes have the capacity of fusing themselves directly with bacterial membranes. This leads to a burst-release of high dose of loaded antibiotics into the bacteria, overwhelming the efflux pumps and overcoming this bacterial resistance mechanism [26]. NPs enable free drug “anti-virulence” therapy. In comparison to traditional antibiotics, nanoparticles are less likely to develop drug resistance, since there is no bactericidal effect, but rather promoted physical and non-specific structural disruption of bacterial membranes, that ultimately led to an increased permeation and bacterial cell death. This process is less likely to elicit resistance development [31]. Furthermore, some anti-virulence platforms include monoclonal antibodies, anti-sera, small-molecule inhibitors or small polymers [26].

### Nanoparticles for bacteria recognition

The recognition of bacteria by detecting specific elements with NPs, which would act as biosensors, is one of the most innovative and emerging strategies to rapidly recognise and treat an infection. The large surface area of nanoparticles allows, not only the increase of the contact between loaded drug and bacterial film, but also a more efficient recruiting of bacterial antigens. Thus, different strategies have been explored in that sense.

#### Antibody-based strategies

NPs surface modification with specific antibodies comprises the most commonly used bio-recognition approach to capture pathogens cells due to their versatility and their high affinity to different bacteria elements. Polyclonal, monoclonal, and engineered antibody fragments are the antibodies employed in immunology-based assays in bacterial infections [32].

#### Aptamer-based strategies

Aptamers are single stranded nucleic acids which offer many advantages over antibody-based strategies. These advantages include their chemical stability, low-cost, and large-scale synthesis process. Moreover, due to their small size (between 3 and 5 nm), aptamers exhibit a remarkable binding affinity against different bacterial cell elements, thus resulting in a significant reduction of the detection limit [32]. Systematic Evolution of Ligands by Exponential enrichment (SELEX) technique is commonly used in the design of aptamers for a huge variety of bacteria [33]. This technique is based on the identification and amplification of an effective aptamer sequence, which will be conjugated with -COOH –SH and -NH_2_ groups and immobilize onto NPs surface.

#### Electrostatic interaction-based strategies

Positively charged nanoparticles can bind to negatively charged protein surfaces, such bacteria cell membrane, through electrostatic interactions. Several type of nanoparticles, such as chitosan nanoparticles, gold nanoparticles, graphene oxide nanoparticles or carbon nanotubes, have been reported to interact with bacteria elements, such enzymes, and inhibiting their activities. Moreover, due to the reversible characteristic of electrostatic interactions, this recognition’s process can be used as an indirect indicator of bacterial cell concentration [32].

#### Bacteriophage-based strategies

Bacteriophages are basic bacteria-infecting viruses comprised by a protein capsid evolving of nucleic acids and tail fibres. These tail fibres are used as recognition elements, which can specifically bind to bacterial surface receptors. The most important characteristic of bacteriophages is their ability to distinguish inactive versus viable host cells, since they can only replicate within alive bacterial cells [34]. In addition, their synthesis and purification processes are relatively easy and inexpensive, together with their medium size (ca. 100 nm), making them a promising biosensoring platform to be combined with NPs for bacteria detection [32].

### Nanoparticles to protect and improve drug effectiveness

The targeting and recognition of specific bacterial structures is not the only strategy to improve antibacterial therapies using nanotechnology. The encapsulation of available antibiotics to improve their properties, as well as new molecules with bactericidal potential, is one of the main objectives of NPs. Likewise, the preservation of the physicochemical properties of encapsulated compounds, as well as an increase in their stability throughout their storage and administration in vivo, are fundamental characteristics provided by nanocarriers that provide a guarantee of success in therapies.

Nitric oxide (NO) is one of the compounds that as raised much interest in the last years because of its antibacterial properties. NO has been recently identified as a key regulator of bacterial biofilm dispersion, but its reduced water solubility, short half-life and extremely reactiveness involve a challenge to exploit its therapeutic potential [35]. In that sense, Duong et al*.* developed two different NO-loaded controlled drug delivery systems: (i) functional gold NPs and (ii) core cross-linked star polymers to improve the stability of NO during the storage and perform a controlled release [36, 37]. Both systems were exposed to *Pseudomonas* biofilms and NO dispersant activity was evaluated. In both cases, nanosystems increased NO stability, showed a slow and controlled delivery of NO, and exhibited a great efficacy in preventing both cell attachment and biofilm formation, with no signals of toxic mechanisms.

Similarly, another interesting study explored the co-encapsulation of NO and gentamicin in poly-oligo(ethyleneglycol) methyl ether methacrylate NPs as a potential antibacterial tool against bacterial biofilm and planktonic cultures [38]. Gentamicin is an aminoglucoside widely used in several bacterial infections, but with serious ear and kidney toxicity. In this study, authors founded that developed NPs were able to perform a controlled release both agents simultaneously, and exhibited synergistic effects. The viability of planktonic cultures and *Pseudomonas* biofilm was reduced by more than 95% and 90%, respectively, whereas free drugs treatments alone resulted in less than 20% of decrease in biofilm viability.

Another antibacterial strategy exploited with nanotechnology is the increase of the temperature in the infected area. It has been described that this increase can induce a detachment of bacteria biofilms and reduction of biofilm biomass. In that sense, Nguyen et al*.* developed iron oxide NPs to disrupt the bacterial biofilm through a NPs-mediated hyperthermia [39]. Upon an exposure to a magnetic field, developed NPs induced the detachment of biofilm cells and reduced by 2-log the number of colony forming units (CFUs) in both biofilm and planktonic phases in combination with gentamicin treatment.

All these findings together may find broad applications across a range of clinical and industrial settings related to bacterial infections management.

## Polymers as a new generation of antibacterial agents

In the last decade, mainly due to the rise of antibacterial drugs resistance, there has been an increase in the search of new compounds able to act against these microorganisms. In this context, antimicrobial polymers (AMP) have emerged as a promising alternative in this field [40]. As described above, polymers by themselves have shown to be promising therapeutic agents against multiple antibiotic resistant bacteria. These materials were designed to mimic the chemical structure of antimicrobial peptides, which are part of the human immune system and are responsible of the protection of the host against different pathogen [41]. Hydrophobicity and cationic charge have been described as the most important properties of these materials to possess antibacterial activity. These characteristics lead to an accumulation of these polymers onto the cell membranes, creating pores on the bacterial structure, thus finally killing them [42].

The composition and distribution of the monomers in the polymer chain have been also described to possess a significant impact in the antibacterial activity. Some studies have pointed out that the incorporation of hydrophobic and amine groups in the terminal positions of the polymer chain allows to vary its hydrophobicity and lead to adjust the extent of bacterium membrane disruption, which in turn governs the antimicrobial activity [43, 44]. Furthermore, it has been also described that the incorporation of oligoethylene glycol in the polymeric structure prevent the complexation of AMP with proteins, which significantly contributes to maintain antimicrobial efficacy [44]. Likewise, polymer architecture also influences in its bactericidal effect. It has been described that polymer longer chains are more bacteriostatic against Gram-negative bacteria, whereas polymer shorter chains have less haemocompatibility. Moreover, hyperbranched polymeric structures possess similar antimicrobial activity compared to the short linear random copolymers with no hemagglutination [45].

Due to their inhering chemical structure, AMP can either promote antibacterial effect or serve as matrix to improve the efficacy of loaded antibiotics [22]. Synergistic effect between AMP and antibiotics have been described by several authors. Thus, Namivandi-Zangeneh et al*.* recently developed an antimicrobial platform composed by a potent synthetic AMP (oligoethylene glycol, ethylhexyl, and primary amine groups) and two antibiotics, colistin and doxycycline [46]. This study reveals the improvement.

of the bacteriostatic efficacy of both AMP and antibiotics when administered together. Moreover, this synergistic combination reduces the rate of resistance development compared to free compounds alone. Similarly, authors recently developed another platform where this AMP was combined with essential oils, carvacrol and eugenol [47]. Essential oils are natural antimicrobial agents produced by many plants. As it was expected, co-administration of these compounds significantly improves the efficacy against *P. aeruginosa* biofilms compared to free compounds. In this case, the synergistic bactericidal activity was attributed to the targeted delivery of carvacrol and eugenol, driven by the electrostatic interaction between positively polymer and negatively charged bacteria.

Ultimately, the versatility in the design of polymers has allowed to exploit all these advantages and obtain different polymeric structures with antimicrobial activity.

### Polymers mimicking natural peptides

Natural peptides are commonly produced as an innate immune response against bacterial infections [22, 48]. The mechanism of action use to be non-receptor dependent and use to involve an interruption of internal cellular function [49, 50]. However, the clinical application of these peptides possesses several limitations because of their potential toxicity to the host, reduced half-life caused by protein binding, and susceptibility to degradation by host proteases [22]. To overcome these disadvantages, in the last years, a variety of polymers have been designed to simulate these natural peptides. Thus, many studies have described synthetic AMP mimicking peptides against most common human bacteria, such *S. aureus*, *E. faecium* or *P. aeruginosa* [22]*.* These synthetic AMP, such brilacidin or idolidicin variants, can act as bacteriostatic or bactericidal compounds with a reduced toxicity to the host, some of them even reaching phase II of clinical trials [51].

### Polymers enclosing phosphor- and sulfo- derivatives

Other option of polymer strategy against bacteria are polysulphonium and polyphosphonium. Their mechanism of action is similar to those polymers which enclose quaternary ammonium in their structure, inducing a bacteria cell wall damage leading to their death [22]. These kind of matrices have been restricted to alkyl and aryl derivatives, which exhibit both hydrophobic and hydrophilic domains, a required feature to possess antimicrobial activity [52].

### Phenol and benzoic derivative polymers

Aromatic compounds, such as benzoic acid or phenol group, have been described to possess antimicrobial properties, and their incorporation in the synthesis of novel polymers grants them the desired antimicrobial activity. Their mechanism of action is also related to the bacteria cell membrane breakdown, and are commonly used as environmental antibacterial agents. Thus, a few decades ago, some authors already explored the synthesis of vinyl polymers containing benzoic or phenol residues as terminal groups to evaluate their effectiveness against *S. aureus* and *P. aeruginosa,* demonstrating their efficacy against this pathogens [53].

### Halogen-derivatives enclosing polymers

Antimicrobial properties of halogens are a well described mechanism that has been used in the design of most common antimicrobial agents, such chloramphenicol or vancomycin (which contain chlorine), or fluoroquinolones (which contains fluorine) [54]. Polymers containing halogens combine the antimicrobial properties of both structures, thus leading to an improvement of therapeutic efficacy. Some examples of this strategy are Quaterfluo®, a polymeric fluorine containing antimicrobial surfactants, cationic fluorinated polymer emulsions, polymers contain *N*-halamines, and halogenated polymers containing antimicrobials agents, such as ciprofloxacin [52, 55–57]. In the latter case, authors demonstrated that the incorporation of ciprofloxacin into the polymeric acidic matrix of amorphous solid dispersions, such Carbopol or Eudragit L100, improved the solubility of ciprofloxacin both in water and in simulated intestinal fluid. Moreover, these formulations improve the minimal inhibitory concentration of ciprofloxacin in *S. aureus*, *E. coli*, *K. pneumoniae* and *P. aeruginosa* cultures [56].

### Amphiphilic antibacterial polymers

It has been demonstrated that polymer structures as well as their physicochemical properties, such as their molecular arrangement, molecular weight or ratio of amphiphilic composition, are the most important determinants of these materials which significantly compromise their selectivity and antimicrobial potency [22]. The ideal amphiphilic antibacterial polymer should possess a low molecular weight and low-level of lipophilicity, harbouring a cationic arm to incur adequate antibacterial activity with a minimum haemolysis activity towards red blood cells [58]. Some studies have demonstrated that AMP-mimicking polyurethanes, with a lower hydrophobic regions and higher cationic strength, show higher bactericidal effect against *E. coli* with a lower haemolysis grade [59]. Likewise, degradable properties of AMP are instrumental for their antibacterial potency. Thus, changes on amine functionality and monomer composition of AMP to control their degradation rate could enhance the lifespan of the antimicrobial activity [60].

### Organometallic polymers

Organometallic polymers are characterized by polymeric matrix containing metals bonded to at least one organic molecule carbon by coordination bonds, π-bonds or by σ-/π-bonds to other elements [22]. One of the most used organometallic polymers are the silver-containing polymers, which have a higher antimicrobial activity in their solid form and have proven to be effective against most common bacterial infections, such *P. aeruginosa, E. coli*, *A. niger* or *S. aureus* [52]. Due to the environmental risk of metals compounds, the use of this kind of materials is compromised. However, a recent study carried out by Awad et al*.* explored the development of an eco-friendly silver-polystyrene nanocarrier using an extract of orange peel to reduce silver nitrate before creating the polymeric matrix [61]. Nevertheless, this kind of material requires further investigation to ensure their environmental safety.

## Recent polymeric nanoparticles against most common human bacterial infections

### Polymeric nanoparticles for pulmonary infections

Respiratory infections, specifically lower tract lung infections, are a leading cause of morbidity and mortality worldwide. In 2016, lower respiratory infections (LRI) caused 2,377,697 deaths in people of all ages, being the adults older than 70 years the cohort of population with a higher risk. *Streptococcus pneumoniae* was the leading cause of LRI morbidity and mortality globally, contributing to more deaths than all other aetiologies combined [62]. According to WHO, pneumonia accounts for 15% of all deaths of children under 5 years old, killing 808 694 children in 2017 over the world [1]. Bacteria related to lung infections show a high resistance to antibacterial drugs, which has promoted a need to find new alternatives to solve this problem. Nanotechnology has shown to be a promising alternative to improve the success of therapy in these diseases (Table 2).

**Table 2:** Selected relevant pre-clinical assays based on novel drug-loaded polymeric nanoparticles for the treatment of bacterial pulmonary infections

Bacteria	Loaded molecule	Polymeric matrix	Surface modifications	Dose	Admin. route	In vitro/In vivo Model	Results	Ref
*Mycobacterium tuberculosis*	–	Chitosan	Tri-mannose	100 µg/m	Cell exposure	A549 cell line Hep G2 cell line	Both un-grafted and grafted PNPs are similarly internalized by macrophages They profoundly remodel the response of *M. tuberculosis*-infected macrophages mRNA sequencing shows nearly 900 genes to be differentially expressed due to tri-mannose grafting (which are enriched for pathways involved in cell metabolism)	Coya et al. [66]
*Chlamydia psittaci*	C. *psittaci* Ags	Chitosan	–	80 μL PNPs-Ags	I.m I.n	BALB/c mice	PNPs-Ags mediate stronger humoral and mucosal responses PNPs-Ags immunization remarkably reduces bacterial load and the degree of inflammation in the infected lungs Furthermore, PNPs-Ags vaccination inhibits *C. psittaci* disseminating to various organs in vivo	Li et al. [70]
*Mycobacterium tuberculosis*	H1 Ag	PLGA	–	0.5 mg/mL	Cell exposure	THP-1 cell line	H1-PNPs are efficiently internalized by the THP-1 human macrophages Immunized mice show significant increase in the production of total serum IgG, its isotypes and inflammatory cytokines levels, compared to H1 alone H1 NP–vaccinated mice display significant reductions in lung and spleen bacillary load, and prolonged survival	Malik et al. [67]
				50 μg PNPs/mouse	I.p	C57BL/6 mice		
*Pseudomonas aeruginosa*	PopB/PcrH	PLGA	–	20 µL PNPs / mouse	I.p	FVB/N mice	PNPs-immunized mice show 3–fourfold higher Th17 responses both in the lung and in the spleen compared to mice immunized with empty PNPs or PopB/PcrH alone PNPs-immunized mice show significantly lower bacterial counts in the lungs and improved survival	Schaefers et al. [64]
*Pseudomonas aeruginosa*	Tobramycin	Alginate / Chitosan	Dornase α DNase	250 μg/mL	Injection	Galleria mellonella	Survival rates of 90% after injection of PNPs A treatment with NPs prior to infection provides a longer antibiotic protection DNase functionalization leads to a DNA degradation and improved NPs penetration Tobramycin NPs both with and without DNase functionalisation, exhibits anti-pseudomonal effects	Deacon et al. [65]

*A549* human alveolar lung cell line, *AMPs* antimicrobial peptides, *C. psittaci Ags Chlamydia psittaci* antigens, *H1 Ag* bivalent antigen of *Mycobacterium tuberculosis* Ag85B and ESAT6 proteins, *Hep G2* Human hepatocytes cell line, *PopB/PcrH* *P. aeruginosa* antigens, *THP-1* human monocytic cell line

Among all bacterial infectious diseases associated with *P. aeruginosa*, lung infections are one of the most frequent, commonly as hospital-acquired pneumonia [63]. In that sense, an interesting study carried out by Schaefers et al*.* explored the development of polymeric NPs (PNPs) co-encapsulated with PopB, the *P. aeruginosa* type III secretion system protein, and its chaperon protein PcrH as a vaccine against IL-17 secretion by CD4 helper T cells in acute *P. aeruginosa* pneumonia [64]. This vaccine was administered intranasally in an in vivo mouse model. At the end of the study, authors found that PNPs-immunized mice exhibit 3–fourfold higher Th17 responses both in the lung and in the spleen compared to those immunized with free PopB/PcrH. Similarly, Deacon et al. explored the potential of PNPs against *P. aeruginosa* lung infections, in this case in comorbidity with cystic fibrosis. The aim of this study was the improvement of tobramycin bioavailability by its incorporation in PNPs. Tobramycin is an aminoglycoside with a limited effectiveness because of its inability to penetrate the thick DNA-rich mucus in the lungs, thus leading to low antibiotic exposure to resident bacteria. The evaluation of the nanocarrier effectiveness was performed in a fly in vivo model of *P. aeruginosa* infection [65]. Obtained results demonstrated that PNPs treatment double the survival rates, achieving 80%, whereas free drug achieves only 40%. When PNPs are functionalized with DNase, a significant DNA degradation in the mucus layer is observed, which is correlated with the enhanced PNPs penetration, thus finally leading to an increased anti-pseudomonal effect.

Tuberculosis infection is the deadliest disease caused by a single infectious agent, *M. tuberculosis*, ahead of malaria and HIV. According to WHO report, there were 10.4 million tuberculosis cases in 2016 and 1.7 million people dead worldwide [1]. Due to the high drug resistance that *M. tuberculosis* presents and the severity and extent of the pathology, a need has arisen to find new therapeutic alternatives against this pathogen. To overcome the limitations encountered in currently available treatments against *M. tuberculosis* lung infections, Coya et al*.* designed mannose functionalized PNPs to improve the response of innate immune human cells [66]. The ability of PNPs to promote inflammation processes could be useful against certain pathogens, but long-term inflammatory responses could be detrimental to the host. Because of that, authors chose the surface modification of PNPs with mannose in order to decrease their pro-inflammatory properties. Authors found that developed PNPs are efficiently internalized by human macrophages and profoundly remodel the response of *M. tuberculosis*‑infected ones. Thus, grafting ligands at the surface of PNPs may be a promising strategy to modulate cell metabolism by immune response route and improve the efficacy of loaded molecules. Similarly, a recent study carried out by Malik et al*.* explored the encapsulation of the bivalent H1 antigen, a fusion of *M. tuberculosis* Ag85B and ESAT6 proteins, in PNPs to investigate its role in immunomodulation and protection in a mice model of *M. tuberculosis* lung infection [67]. In this case, developed PNPs are also efficiently internalized by the THP-1 human macrophages and the immunized mice exhibit a significant increase in the production of total serum IgGs. Moreover, in protection studies, immunized mice display significant reductions in lung and spleen bacterial load and prolonged survival.

*Chlamydia psittaci* infections are commonly transferred to humans via the inhalation of contaminated aerosols originating from excretions from infected animals. A recent study reported that *C. psittaci* lung infection may contribute to increase the risk of co-infection with other pathogens, including the flu virus, and has highlighted to be a one of the causes of community-acquired pneumonia [68, 69]. Related to that, Li et al. developed a novel immunization strategy, simultaneous intranasal and intramuscular administration of PNPs coated with *C. psittaci* antigens to determine the different types of immune response and their protective role in an in vivo model of lung infection [70]. Obtained results showed that developed PNPs promote a strong immunity response by producing meaningfully high levels of IgG and secretory IgA antibodies. In addition, in vivo results exhibited that PNPs vaccine inhibits *C. psittaci* dissemination to different organs, as well as a reduction of bacterial loading and the degree of inflammation in the infected lungs.

### Polymeric nanoparticles for oral cavity infections

Oral cavity infections, mainly caused by bacterium biofilm formation in different oral structures, involve a major public health and economic burdens worldwide. Oral biofilms are responsible for causing tooth decay and dental caries, and present a high prevalence in humans (> 90% of adult population and (> 30% of schoolchildren over the world) [71]. Just in U.S., annual expenditures related to oral cavity infections interventions exceed $120 billion [72]. In addition, drug retention in the oral cavity remains a major challenge, thus limiting the clinical practice incorporation of many anti-biofilms active compounds [73]. All of these reasons have triggered the development of various PNPs in recent years to treat oral cavity infections (Table 3).

**Table 3:** Selected relevant pre-clinical assays based on novel drug-loaded polymeric nanoparticles for the treatment of bacterial oral cavity infections

Bacteria	Loaded molecule	Polymeric matrix	Surface modifications	Dose	Admin. route	In vitro/In vivo model	Results	Ref
*Streptococcus mutans*	–	Chitosan	–	15–45% of NPs	Incubation	*S. mutans* ATCC 25,175	PNPs significantly decrease the cell viability of both microorganisms	Ikono et al. [77]
*Porphyromonas* g*ingivalis*	–	PLGA	BAR	0.7 μM of NPs	V.o	BALB/cByJ mice	Treatment of infected mice with PNPs reduce bone loss and IL-17 expression almost to the levels of sham-infected mice and to a greater extent than treatment with an equimolar amount of free BAR	Mahmoud et al. [80]
*Streptococcus mutans*	Farnesol / thonzonium bromide	p(DMAEMA)	–	0.125–64 μg/ml	Incubation	*S. mutans* UA159 serotype c	Farnesol PNPs reduce total biomass by disrupting insoluble glucan formation and increase NPs-cell membrane localization Thonzonium bromide NPs reduce biofilm cell viability by ~ 6 log CFU	Sims et al. [78]
*Staphylococcus aureus* *Escherichia coli*	–	PEG-PAE	Triclosan / Salivary proteins	200 µl of NPs	Incubation	*S. aureus* or *E. coli* biofilms	In vitro*, *in vivo and ex vivo results show that PEG-PAE-Triclosan yield better eradication efficacy towards a MDR *S. aureus, E. coli* and oral streptococcal biofilms than free Triclosan	Liu et al. [79]
					I.v	BALB/c nude mice		
*Porphyromonas gingivalis* *Lactobacillus lactis Streptoccocus mutans Streptoccocus gordonii* *Streptoccocus sobrinus*	Calcium zinc doxycycline	2-hydroxyethyl methacrylate, ethylene glycol dimethacrylate and methacrylic acid	–	0.1–10 mg/mL	Incubation	*P. gingivalis* 33,277 *S. mutans* 700,610 *S. sobrinus* 33,478 *S. gordonii* 10,558 *L. lactis* 12,315	Dox-PNPs are the most effective antibacterial material, followed by Ca-PNPs, Zn-PNPs and finally the non-doped PNPs *P. gingivalis*, *S. mutans* and *L. lactis* are the most susceptible bacteria, being *S. gordonii* and *S. sobrinus* the most resistant to the tested PNPs	Toledano-Osorio et al. [82]

*BAR* a peptide derived from *Streptococcus gordonii,* *p(DMAEMA)* Poly(dimethylaminoethyl methacrylate), *PEG-PAE* poly(ethylene)glycol-poly(b-amino esters)

*Streptococcus mutans* and *Candida albicans* are the two pathogens which are commonly associated to aggressive dental caries, most frequently affecting children from developing countries [74–76]. Importantly, both bacteria interact synergistically in forming dual-species biofilms, which hardly complicate their treatment. Since chitosan NPs have proven to be effective against a broad spectrum of pathogens, Ikono et al. evaluated their effect on dental caries-associated to *S. mutans* and *C. albicans* biofilms [77]*.* Obtained results showed that PNPs significantly decrease the cell viability of both pathogens in a concentration dependent manner. Moreover, although biofilm mass is not reduced in the first hours with these PNPs, a greater inhibition of biofilm growing is observed at 18 h of chitosan NPs incubation in the site of action. Similarly, Sims and colleges explored the potential of PNPs as a therapeutic tool against *S. mutans* oral biofilms. In this case, authors developed two novel pH-responsive PNPs containing farnesol (a hydrophobic antibacterial drug) and thonzonium bromide (a highly potent, FDA-approved antibacterial drug), respectively [78]. In this case, authors found that both developed PNPs markedly amplify the anti-biofilm activity of loaded molecules, showing reductions of ~ 2 to 6 log CFUs. Furthermore, farnesol-loaded PNPs are able to reduce total biomass by disrupting insoluble glucan formation. All these findings open a new window for the local treatment of dental caries caused by *S. mutans* biofilms.

Another interesting study related to oral biofilms was performed by Liu et al*.* who developed micellar PNPs which possess pH adaptivity to self-target to the acidic environment of bacteria biofilm. This carrier was composed by a PEGylated poly(β-amino esters) matrix, surface conjugated with triclosan, a potent bactericidal and fungicidal agent. Its effectiveness was evaluated both in in vitro and in vivo models of MDR *S. aureus* and *E. coli* oral biofilms[79]. In vitro results showed that PEG-PAE-triclosan are more efficient in killing MDR *S. aureus, E. coli* and oral streptococcal biofilms than free triclosan. Furthermore, in vivo assays showed that these PNPs also yield better eradication efficacy towards a MDR *S. aureus*-infection compared to free triclosan, exhibiting a bacterial killing at 30–40 fold lower triclosan-equivalent concentrations than achieved by triclosan in solution in the ex vivo analysis.

Mahmoud et al. also explored the potential of PNPs in the therapeutic approach of bacterial oral infections, in this case of *Porphyromonas* g*ingivalis,* one of the most common periodontal pathogens in humans [80]. It has been shown that the interaction between commensal streptococci and *P.*g*ingivalis* significantly promotes the colonization of the oral cavity by *P.* g*ingivalis.* This study describes the development of PLGA NPs surface-modified with BAR, a peptide derived from *Streptococcus gordonii*, which has previously shown to effectively inhibit the adherence of *P.* g*ingivalis* to streptococci, thus reducing *P.* g*ingivalis* periodontitis. Since BAR is mainly effective when biofilm is not yet established, the aim of this work was to improve the effect of BAR by PNPs targeting and bioaccumulation, to significantly disrupt an already established *P.* g*ingivalis /S. gordonii* biofilm [81]. Obtained results showed that developed nanocarrier significantly improve bone loss and IL-17 expression (analysed parameters of periodontal infection) of infected animals. Moreover, PNPs shown to be safe for gingival keratinocytes and red blood cells both in vitro and in vivo. Thus, this therapeutic alternative highlights the potential of PNPs as a biocompatible platform for local translatable oral biofilm applications.

Similarly, Toledano-Osorio et al*.* developed three different PNPs loading respectively zinc (Zn), calcium (Ca) and doxycycline, and evaluated their efficacy against several bacteria involved in oral infections, such *P.* g*ingivalis, S. gordonii* or *S. mutans,* among others[82]. Authors demonstrated that developed PNPs are able to reduce the bacterial viability, been the doxycycline PNPs the most effective, reducing viability almost up to 99%, followed by Ca PNPs and Zn PNPs. Furthermore, they also found that the most PNPs-susceptible bacteria are *P. gingivalis, S. mutans* and *L. lactis*, and being *S. gordonii* and *S. sobrinus* the most resistant.

### Polymeric nanoparticles for gastrointestinal tract infections

Causes of gastrointestinal infections include viral, bacterial, parasitic and fungal pathogens. They vary among different geographical regions, and are compromised by co-morbidities and host immune status [1]. Bacteria are responsible for 20–40% of diarrhoeal episodes worldwide, thus contributing to high rates of childhood mortality in developing regions, and substantial morbidity and economic losses in developed regions [83]. Due to the severity of these diseases, many authors are been recently analysing the potential of PNPs to improve the pharmacological properties of existing antibiotics and/or to explore novel therapeutic alternatives (Table 4).

**Table 4:** Selected relevant pre-clinical assays based on novel drug-loaded polymeric nanoparticles for the treatment of bacterial gastrointestinal infections

Bacteria	Loaded molecule	Polymeric matrix	Surface modifications	Dose	Admin. route	In vitro/In vivo model	Results	Ref
*Helicobacter pylori*	CLR	PLGA	AGS cells/PEG	200 μL	Incubation	*H. pylori* Sydney strain 1	CLR-loaded AGS-NPs demonstrate higer efficacy when compared with the free drug as well as a non-targeted NPs	Angsantikul et al. [91]
				30 mg/kg	O.g	C57BL/6 mice		
*Vibrio cholerae*	–	PLGA	GM1 / PEG	1 mg/mL	Incubation	*V.cholerae* N16961 ATCC cells	GM1-NPs show to function as toxin decoys by selectively and stably binding cholera toxin, and neutralizing its actions on epithelial cells in vitro and in vivo	Das et al. [84]
				250 μg/mL	Intestine exposure	C57BL/6 mice		
*Mycobacterium marinum*	–	PLGA	–	5 μL of NPs	Oral intubation	Zebrafish	NPs are rapidly taken up in the intestine and transported to the liver and spleen	Lovmo et al. [93]
*Campylobacter jejuni*	CpG ODN	PLGA	–	5/25 µg of NPs	Oral treatment	Chickens	The microbiota of CpG ODN-NPs-treated chickens exhibits higher microbial diversity and lower numbers of *Campylobacter* than untreated-chickens	Taha-Abdelaziz et al. [85]
*Salmonella typhimurium*	cryptdin	Chitosan	–	10/15 μg of NPs	O.G	BALB/c mice	Infected mice treated with NPs show 83% survivability and approximately 2 log unit reductions in the bacterial load in the tissues versus 100% mortality observed with the free peptide	Rishi et al. [89]

*AGS cells* plasma membranes of gastric epithelial cells, *ATCC* Human HCA7 colon cancer cells, *CLR* clarithromycin, *CpG ODN* CpG oligodeoxynucleotide, *GM1* monosialotetrahexosylganglioside, *HCQ* Hydroxychloroquine

As explained above, *V. cholerae* is the bacterium that causes cholera in humans and the most common cause of bacterial diarrhoea worldwide [14]. As happens with other bacterial infections, the appearance of MDR has promoted an urgent need to develop alternative treatments against *V. cholera*. In that sense, Das and colleges designed surface modified PLGA NPs with PEG and GM1, a key host receptor for choler toxin, as a recruitment and clearance tool of enterotoxin [84]. Obtained results demonstrated that these PNPs are able to selectively and stably binding cholera toxin, thus neutralizing its actions. Moreover, developed PNPs attenuate the production and fluid responses of epithelial 3′, 5′-cyclic adenosine monophosphate both in in vitro and in vivo models of *V. cholera* infection.

Gastroenteritis is one of the most common gastrointestinal disorders in humans, and is caused by a few pathogens, among which *C. jejuni* stands out. Farm chickens are an important reservoir of *C. jejuni* and although it does not cause clinical symptoms in these animals, it can be transmitted to humans by consumption of undercooked poultry chicken meat contaminated by intestinal contents. Reducing the *C. jejuni* colonization of gastrointestinal tract of farm chickens would lead to a reduction of incidence of human gastroenteritis. Related to that, Taha-Abdelaziz et al. developed PNPs containing CpG oligodeoxynucleotide (ODNs), a short synthetic DNA sequence highly prevalent in bacterial DNA, in order to evaluate their potential effectiveness in reducing *C. jejuni* burden in chickens [85]. ODNs have shown to bind to and activate Toll-like receptor 9, which initiate an innate immune response that subsequently promotes the development of an adaptive immunity against the bacteria to which originally belong [86]. The authors found that developed PNPs are able to reduce the intestinal burden of *C*. *jejuni* in chickens, protect the intestinal epithelium and improve systemic immune responses of these animals. The administration of these PNPs together with *C. jejuni* lysate enhances the proliferation of specific microbial groups that reduce the colonization of *C. jejuni*.

Another bacteria pathogen associated with gastroenteritis and diarrhoea disorders is *Salmonella* spp., which has become a major public health problem worldwide [87, 88]. MDR issues also compromise antibiotic treatment of *Salmonella* infections, thus efforts in identifying an alternative therapeutic approach against *Salmonella* are gaining much interest. Cryptdins, Paneth cell peptides, have been reported to own bactericidal activity against several intestinal pathogens. To improve their pharmacological properties, Rishi and colleges developed cryptdin-loaded chitosan NPs to test their effect in a mice model of *Salmonella typhimurium* gastrointestinal infection [89]. Developed PNPs significantly increase the survival ratio of treated mice, whereas 100% mortality was observed with the free peptide treatment. Furthermore, cryptdin-loaded PNPs are able to decrease the levels of oxidant molecules, increase the level of antioxidants ones, thus all together has been the first pre-clinical report of cryptdin effectiveness by its suitable encapsulation.

*Helicobacter pylori* is a gram-negative, helical bacillus-shaped bacterium that lives in the human stomach exclusively. Although, infected individuals may never have any symptoms, *H. pylori* infection can produce inflammation of the gastric mucosa that can progress leading to gastritis, peptic ulcer, and mucosa-associated lymphoid tissue lymphoma, thus posing a significant healthcare burden worldwide [90]. Related to that, Angsantikul et al*.* recently developed clarithromycin-loaded PLGA surface modified NPs with PEG and molecules of plasma membranes of gastric epithelial cells [91]. Nowadays, triple therapy based on a combination of clarithromycin, proton pump inhibitor and an antibiotic, such metronidazole or amoxicillin, is recommended for the treatment of *H. pylori* infection, but its recent mutations and subsequent drug resistance significantly compromised the success of this therapeutic option [92]. In this study, authors found that, as it was expected, developed PNPs mostly accumulate on the bacterial surface in an in vitro model of *H. pylori*. Moreover, surface modified-PNPs exhibit a higher therapeutic efficacy in mouse model of *H. pylori* infection, when compared with the free clarithromycin and to the non-targeted PNPs. Overall, these results illustrate the potential of using natural host cell molecules to functionalize PNPs for targeting and delivering of antibiotics against pathogens that colonize on that host cells.

Finally, to analysed the biodistribution of PNPs in an in vivo infection model, an innovative study carried out by Lovmo et al*.* evaluated the absorption and interactions of PLGA NPs with epithelial cells and the mucosal immune system of a zebrafish in vivo model of *Mycobacterium marinum* infection, an opportunistic bacteria in humans [93]. By fluorescence imaging, authors found that PNPs, as well as bacteria, are rapidly taken up in the intestine and mainly transported to the spleen and liver. Furthermore, in these organs, both PNPs and *M. marinum* bacteria cells are largely localized to leukocytes, presumably macrophages.

### Polymeric nanoparticles for skin infections

Bacterial skin infections e.g. folliculitis, impetigo, furunculosis, and wound infections, among others, are the most common type of skin infections [94]. Recent studies have explored the potential of PNPs as a promising therapeutic alternative for skin infections (Table 5). The major problem resulting from wound infections caused by MRSA is the colonization of other organs, which lead to an invasive and deep infection, resulting in systemic bacteraemia and significant morbidity and mortality. Related to this, Hasan et al*.* developed mixed PNPs based on PLGA/PEI with loaded clindamycin, a semisynthetic antibiotic, derived from lincomycin, effective against aerobic gram-positive cocci and anaerobic gram-negative bacilli. The developed nanocarrier was tested in a MRSA culture and ICR mice model of cutaneous wound infection [95]. Authors found that developed PNPs are able to efficiently bind to the MRSA surface, thus enhancing the bactericidal activity of clindamycin. Moreover, clindamycin-loaded PNPs significantly accelerate the healing and re-epithelialization of wounds in the in vivo assay, without compromising the healthy fibroblast cells activity.

**Table 5:** Selected relevant pre-clinical assays based on novel drug-loaded polymeric nanoparticles for the treatment of bacterial skin infections

Bacteria	Loaded molecule	Polymeric matrix	Surface modifications	Dose	Admin. route	In vitro/In vivo model	Results	Ref
*Staphylococcus aureus*	clindamycin	PEI/PLGA	–	0.1–0.5 mg/mL	Incubation	MRSA	Cly/PPNPs enhance bactericidal efficacy against MRSA compared with the Cly/PNPs Cly/PPNPs significantly accelerate the healing and re-epithelialization of wounds in infected mice Both NPs are harmless to healthy fibroblast cells	Hasan et al. [95]
				0.5 mg/ml	Topically applied	ICR mice		
*Pseudomonas aeruginosa*	PDH	PLGA	–	125 μL of PNPs	Incubation	Biofilms PAO1	The optimal formulation disperses biofilms and exhibits enzymatic activity	Han et al. [96]
*Staphylococcus epidermidis*	Propolis	Chitosan	–	100 μg/mL of PNPs	Incubation	*S. epidermidis* strain ATCC 14,990	PNPs effectively disrupt biofilm formation of S. epidermidis and decrease its viability to ~ 25% Gene expression in treated bacteria shows that genes involved in intercellular adhesion such as *IcaABCD*, *embp* and other related genes are significantly downregulated by PNPs exposure	Ong et al. [98]
*Staphylococcus epidermidis*	Imidazolium cations	PLGA	Chitosan	1 mg PNPs/well	Incubation	LIVE/DEAD kit	PNPs show a high antibacterial activity to the bacterial cells under the biofilm	Takahashi et al. [99]

*LIVE/DEAD* LIVE/DEAD BacLight bacterial viability kit (Cat. Num. L-13152), *MRSA* methicillin-resistant *Staphylococcus aureus,* *PDH* pyruvate dehydrogenase

Another pathogen found in chronic wound infections, as well as burn wound infections, is *P. aeruginosa*. Han et al. developed enzyme-encapsulated PLGA NPs and evaluated their effectiveness against biofilms formed by *P. aeruginosa* PAO1 [96]. Selected enzyme was pyruvate dehydrogenase, since it has been previously described that *P. aeruginosa* biofilm are reverted to an antibiotic-susceptible state via pyruvate-depletion induced dispersion [97]. Obtained results showed that developed PNPs extended the activity of this enzyme, thus leading to the dispersion of preformed biofilms.

*Staphylococcus epidermidis*, a common microbiota of human body, can originate opportunistic infections associated with indwelling medical devices. It shows a strong MDR activity, which promotes the need to find new pharmacological alternatives. In this context, Ong and colleges designed chitosan NPs carrying malasyan propolis to evaluate their efficacy against this opportunistic pathogen [98]. Malaysian propolis, obtained from beehives, is a natural product that exhibits significant antimicrobial activity. Authors found that developed PNPs are able to effectively disrupt *S. epidermidis* biofilm formation and decrease the bacteria viability to ~ 25%. Moreover, propolis-loaded PNPs downregulate genes involved in intercellular bacteria adhesion, and exhibit a synergism with ciprofloxacin, rifampicin, doxycycline and vancomycin. Thus, combination therapy represents a way of tackling antibiotic resistance in *S. epidermidis* infections. Similarly, Takahashi et al. evaluated the potential of PNPs as a novel tool against *S. epidermis* biofilm skin infections. In this case, polymeric matrix was composed by PLGA, and imidazolium cations were loaded as active compound [99]. These molecules present some advantages, such as good biocompatibility, high permeability to bacteria cell wall, and significant antimicrobial activity. Thus, by using a LIVE/DEAD BacLight bacterial viability kit, authors demonstrated that developed PNPs possess a high antibacterial activity to the bacterial cells forming the biofilm, establishing a suitable drug delivery system to enhance the potential of polymeric nanocarriers for treating biofilm infections.

### Polymeric nanoparticles for urinary tract infections

Urinary tract infections are the most common outpatient infections, with a lifetime incidence of 50 − 60% in adult women [100]. The prevalence of urinary infection is 0.7% worldwide, being the age, sexual activity and diabetes the main risk factors. The most common pathogen is *Escherichia coli*. Importantly, resistance rates of most common antibiotic drugs of urinary tract are increasing over the years, and are significantly governed by geographical location [101]. For these reasons, in the last years, several nanotechnological platforms have been exploited for the treatment of urinary tract infections (Table 6).

**Table 6:** Selected relevant pre-clinical assays based on novel drug-loaded polymeric nanoparticles for the treatment of urinary tract infections

Bacteria	Loaded molecule	Polymeric matrix	Surface modifications	Dose	Admin. route	In vitro/In vivo model	Results	Ref
*Pseudomonas aeruginosa* *Klebsiella Pneumoniae*	–	Chitosan	–	5 − 100 μg/mL	Incubation	Pseudo 9 HX053 Pseudo 12 HX103 Kleb 1—HX033 Kleb 2—HX077	Biofilm formation of *P. aeruginosa* and *K. pneumoniae* is inhibited at 40 μg/mL of PNPs up to 94 and 92%, respectively	Maruthupandy et al. [111]
*Escherichia coli*	proanthocyanidin	Chitosan	–	200 mg/mL of PAC	Incubation	ExPEC	PNPs decrease the ability of ExPEC to invade epithelial cells in a dose-dependent manner	Alfaro-Viquez et al. [105]
*Escherichia coli*	Ag NPs	–	PVP	1.25–0.039 mg/mL	Incubation	*E. coli* ATCC 25,922	Coated PNPs inhibit the growth of *E. coli* at 0.312 mg/mL Coated NPs imped bacterial growth as early as 8 h at 0.156 mg/Ml	Ashmore et al. [104]
*Staphylococcus aureus* *Escherichia coli* *Pseudomonas aeruginosa* *Proteus mirabilis* *Canddida albicans*	–	Hyaluronic acid	–	NA	Surface immobilization	*S. aureus* *E. coli* *P. aeruginosa* *P. mirabilis* *C. albicans*	Microbial growth is hampered by 85% compared with unmodified PNPs silicone catheters	Bracic et al. [109]
*Staphylococcus aureus* *Staphylococcus epidermis* *Escherichia coli* *Enterococcus faecalis* *Pseudomonas aeruginosa* *Proteus mirabilis*	Norfloxacin AgNPs	PLGA	–	NA	Surface immobilization	*S. aureus* *S.epidermides* *E. coli* *E. faecalis* *P.aeruginosa* *P. mirabilis*	The polymer films loaded with the two antibacterial agents avoid biofilm formation for at least 2 weeks	Dayyoub et al. [110]
*Escherichia coli* *Proteus mirabilis*	Kanamycin	Chitosan	–	NA	Surface immobilization	*E. coli* MTCC 729 *P. mirabilis* MTCC 425	The PNPs surface-modified ureteral stent shows significantly increased antibacterial activity relative to the surface of an unmodified one	Kumar et al. [106]
*Escherichia coli* *Staphylococcus aureus*	Chlorin e6	Poly(HDDA-co-DEPA)	PEG	100 μl NPs	Incubation	*E. coli* ATCC 8739 *S. aureus* ATCC 6538	In vitro*:* PNPs show significant antibacterial efficacy in vitro with low cytotoxicity In vivo: a significant decline in bacterial cells count occurred in urine after PNPs injection together with photodynamic therapy treatment	Liu et al. [108]
				50 μl of NPs	Bladder injection	BALB/c mice		

*ExPEC *extra-intestinal pathogenic Escherichia coli; *PVP* polyvinylpyrrolidone

*E. coli* is a gram-negative bacillus bacterium that is part of the normal microbiota of the gastrointestinal tract in humans. However, different strains have been shown to acquire genetic mutations that are related to virulence factors [102]. These strains have been associated with gastrointestinal, urinary tract, blood or nervous system infections. The high morbidity, as well as the variety in syndromes and clinical symptoms associated with *E. coli* infections, make this bacterium one of the most versatile pathogens of great relevance to humans [103]. Several studies have evaluated the potential of PNPs as therapeutic tool against *E. coli* urinary tract infections. Related to this, Ashmore and colleges compared the antibacterial efficacy of polymer-coated silver NPs with the non-coated ones [104]. The coating of silver NPs with polymer significantly improves the antibacterial efficacy of the particles. In addition, polymer-coated silver NPs also promote a downregulation of the expression of genes associated with the citric acid cycle and amino acid metabolism, which are involved in the cellular growth of *E coli.* Similarly, Alfaro-Viquez et al. developed proanthocyanidin-chitosan NPs against bacterial colonization of gut epithelial cells by extra-intestinal pathogenic E. coli (ExPEC) [105]. ExPEC are the primary cause of urinary tract infections, of which 20% to 45% have shown resistance to antibiotics [102]. Cranberry proanthocyanidins have widely shown to be effective in the treatment of acute cystitis, which indicate that could be a potential alternative as antimicrobial therapy of ExPEC infections. However, the antioxidant properties of these compounds make them very susceptible to degradation, thus reducing their pharmacological effectiveness. This study revealed that developed PNPs protect proanthocyanidins from oxidation and decrease the ability of ExPEC to invade epithelial cells in a dose-dependent manner.

Co-infections commonly occur in urinary tract. There is a wide variety of bacteria that can colonize human urinary tract and also related medical devices (e.g. catheters or ureteral stents). Kumar et al*.* explored the surface immobilization of kanamycin-loaded PNPs to prevent the bacterial biofilm formation in urethral stents [106]. Kanamycin is an aminoglycoside antibiotic used to treat Gram-negative and some Gram-positive bacterial infections. Recently, is has been shown that combination of antibiotics with chitosan matrices significantly improve the antibacterial activity of these molecules and reduce the resistance of several bacteria [107]. In this study, authors reported that, after a covalent immobilization of developed kanamycin-loaded PNPs onto the surface of an urethral stent, it is observed a significant reduction of *E. coli* and *P. mirabilis* activity, the selected bacteria. Similarly, an interesting work developed by Liu et al*.* explored a therapeutic alternative for urinary co-infections of *E. coli* and *S. aureous*, which involved the combination of antimicrobial photodynamic therapy together with PNPs [108]. The technique was based on the photostimulation of Chlorin e6 encapsulated charge-conversion PNPs by a laser irradiation, which generates the production of reactive oxygen species (ROS) and subsequently killing of pathogenic bacteria under an acidic environment. This innovative technique was evaluated in both in vitro and in vivo models. Obtained results showed that PNPs are able to better recognise Gram-positive (*S. aureus*) and Gram-negative (*E. coli*) bacteria due to the charge interaction, together with a significant in vitro antibacterial efficacy and reduced cytotoxicity. Moreover, in a mouse acute cystitis model, authors found a significant decline in bacterial cells counts in the urine of NPs-treated animals compared to those treated with free drug, as well as in the bladder tissue after sacrifice.

Another interesting study carried out by Bracic et al*.* explored the immobilization of PNPs composed by hyaluronic acid and a lysine-derived biocompatible cationic surfactant in silicone catheters to evaluate their efficacy against five different colonizing bacteria of urinary tract medical devices [109]. In this case, obtained results revealed that that the microbial growth is hampered almost by 85% compared to unmodified silicone catheters. Taking a step further, Dayyoub et al*.* developed silver NPs coated with PLGA carrying norfloxacin, a broad-spectrum synthetic antibiotic almost exclusively indicated in the treatment of urinary tract infections [110]. These PNPs were used to coat polyurethane and silicon sheets. This coating prevents the bacterial adhesion of artificial urine and an in vitro encrustation model for at least 2 weeks.

*P. aeruginosa* and *K. pneumoniae* are not only colonizers of the respiratory tract, but also of the urinary tract. Related to that, Maruthupandy et al*.* recently developed graphene/chitosan NPs against *P. aeruginosa* and *K. pneumoniae* biofilms [111]. With this study authors demonstrated that the viability of both uropathogens is reduced almost 95% when incubated with developed hybrid NPs.

### Polymeric nanoparticles for neuroinfections

Central nervous system (CNS) infections can be caused by different agents (e.g. viruses, bacteria, parasites, fungi) and, in rare occasions, by prions infections. Meningitis and encephalitis are the most common neuroinfections in humans, which involve the inflammation of the whole brain of the meninges, the membranes that surround the brain and spinal cord, producing a high impact on tissue homeostasis [112]. There is a different etiological epidemiology of meningitis and encephalitis in different geographical regions; pathogens vary across different age groups, seasons and geographic locations. Viruses account for most cases of meningitis and encephalitis. Viral meningitis and encephalitis are usually benign and self-limiting, while bacterial meningitis and encephalitis can lead to disastrous damage [113]. The cardinal clinical symptoms and signs are mainly indistinguishable regardless of the inciting pathogen [114].

CNS infections cause significant morbidity and mortality and often require neurosurgical intervention for proper diagnosis and treatment. Regarding to WHO, Africa had the highest pooled incidence of bacterial meningitis (65 cases/100,000 people) [115]. Approximately 304,000 meningitis patients and 77,000 encephalitis patients died all over the world in 2013 comparing to 46,4000 and 92,000 in 1990 respectively [116].

One of the main limitations during bacterial neuroinfections treatment is the inability of antibiotics to surpass the BBB and the blood–cerebrospinal fluid barrier (BCSFB), as well as the emerging of antibiotic resistance. To this end, drug targeting approaches are being recently used to overcome these issues [117]. Getting a local antimicrobial treatment, the concentration of the drug at the site of infection will be improved, as it may modulate drug-pathogen interaction to overcome antibiotic resistance, and enabling 'drug-free' anti-virulence therapy [26]. In this context, many studies have been conducted in order to improve antibacterial and antivirals compounds effectiveness by using of nanotechnology (Table 7).

**Table 7:** Selected relevant pre-clinical assays based on novel drug-loaded polymeric nanoparticles for the treatment of bacterial neuroinfections

Bacteria	Loaded molecule	Polymeric matrix	Surface modifications	Dose	Admin. route	In vitro/In vivo model	Results	Ref
*Streptococcus pneumonia*	Bacitracin A	PLGA	PEG RGV29 P-gP inhibitor	30 mg/kg	I.v	Kunming mice + *S. pneumonia* ATCC49619 and *S. pneumonia* 16,167	In vivo results further demonstrated that PNPs were able to accumulate in brain parenchyma and exhibited high therapeutic efficiencies in resistant PM mouse models with negligible systemic toxicity	Hong et al. [122]
*Pseudomonas aeruginosa*	LPS	Acrylamide and N,N´-methylenebisacrylamide	–	5 mg/kg of NPs	I.c.v	Kunming mice + *P. aeruginosa*	Selective recognition of target bacteria. Significantly strong inhibition of bacterial growth	Long et al. [120]
*Neisseria meningitidis*	CPS-A	Albumin	–	250 µg of NPs	–	DC2.4 cell line	Surface expression of MHC I, MHC II, CD95 and co-stimulatory molecules in dendritic cells were incremented with CPS-loaded PNPs	Gala et al. [119]

*CPS-A* Meningococcal capsular polysaccharide antigen from serogroup A, *LPS* lipopolysaccharides from *P. aeruginosa*, *RVG29* brain-targeted gene

*N. meningitidis* is a leading cause of bacterial meningitis and its capsular polysaccharides the basis for serogroup designation and preventive vaccines [118]. Related to that, Gala et al. recently developed a novel meningococcal nanoparticulate vaccine that slowly release those antigens, and evaluated the possibly antigen depot effect to enhance antigenicity [119]. In order to enhance the antigenicity of the vaccine, various adjuvants were evaluated. Results showed that PNPs enhance dendritic cell maturation and antigen presentation markers MHC I, MHC II, CD40, CD80 and CD86 in this cell line pulsed with meningococcal nanoparticulate vaccine. Similarly, Long et al*.* developed PNPs to carry amphiphilic lipopolysaccharides, derived from *P. aeruginosa* to test their effectiveness in a mice model of meningitis [120]. *P. aeruginosa* has been shown also to infect the brain and spinal cord, and lead to meningitis, brain abscesses, and potentially death [121]. Authors found that developed PNPs promote a selective recognition of target bacteria and exhibit targeting capacity in vivo. Moreover, this nanocarrier presents a strongly inhibited bacterial growth, compared to those controls of the in vitro assays.

The incidence of CNS bacterial infections is associated with the immunosuppressive drug treatments and increased prescription of antibiotics. Immunosuppression is usually followed by immunodeficiency disease, which led to those serious infections and, in case of CNS, BBB compromises the cumulative of antibacterial agents. In this context, Hong et al. developed a PEGylated-polymeric nanocarrier of bacitracin A coated with a specific brain-targeting peptide and a P-glycoprotein inhibitor against Pneumococcal meningitis (122). This study revealed the enhanced BBB-permeability attributed to the synergetic effect of both coating peptides. In vivo results further demonstrated that developed PNPs are able to accumulate in brain parenchyma and exhibit high therapeutic efficiency with negligible systemic toxicity compared to the free drug.

## Conclusions

The rise of the number of individuals affected by bacterial infectious diseases constitutes a significant socioeconomic health burden worldwide and is mainly related to economic and public health problems in developing countries, and nosocomial infections in developed countries. Even when promising therapeutic molecules are available, the appearance of MDR events has established itself as a serious public health problem worldwide. The widespread misuse of antibiotic drugs has promoted genetic mutations and smart evasion mechanisms by bacteria that have led to an important failure in these therapies. As reviewed herein, nanotechnologies applied to medicine—nanomedicine—have opened new therapeutic opportunities for the treatment of bacterial infections. Specifically, state-of-the-art PNPs have demonstrated to possess optimal physicochemical characteristics to become a therapeutic revolution against human bacterial infections. This type of nanocarriers have shown to be safe, biodegradable, biocompatible, easily eliminated and non-toxic for the tissues and organs, offer many advantages for currently available molecules. Moreover, the targeting to a specific organ, the reduction of the adverse effects of many antibiotics, and prolonged accumulation over time in the infected area, altogether represent a breakthrough in this kind of therapy. Furthermore, polymers themselves have shown to be effective against multiple antibiotic resistant bacteria. Thus, the combination of loaded drugs together with polymeric matrices may offer a synergistic approach with innovative outputs and may represent the next step in the treatment of bacterial infections.

Research in the next few years will increasingly focus on the elucidating and better understanding of the molecular mechanisms of bacteria involved in MDR events to re-direct the drugs targets. Whilst PNPs are undoubtedly among the most efficient tools to overcome MDR bacteria problems, clinical translation of nanoparticles-based treatments remains a challenge for the pharmaceutical industry, since nanotechnology has not yet entered mainstream clinical practice. Stringent protocols of validation of in vitro and in vivo protocols are necessary to facilitate translation from bench to the clinical trials. Likewise, challenges for large scale manufacturing require innovation from chemists and engineers and regulatory policies have to be adapted to facilitate access to trials and patients. Thus, despite the huge progress in preclinical research, the translation of these drug delivery systems to clinical practice remains one of the most ambitious challenges in this field, and will be a major focus of most nanomedicine trials in the next decade.

Finally, the social gap between developed and developing countries, which is closely related to the rise of these diseases, is another of the important challenges that we face as a society. The development of a global community structure that ensures access to antibiotic drugs and their correct use, and effective public health measures will represent an advance against bacterial infections worldwide.

## Data Availability

Review Manuscript. Not applicable.

## Abbreviations

AMP: Antimicrobial polymers
BBB: Blood–brain barrier
BCSF: Blood–cerebrospinal fluid barrier
CFUs: Colony forming units
CNS: Central nervous system
ExPEC: Extra-intestinal pathogenic *E. coli*
LRI: Lower respiratory infections
MDR: Multidrug resistant
MRSA: Methicillin-resistant *S. aureus*
NO: Nitric oxide; NPs, nanoparticles
ODNs: CpG oligodeoxynucleotides
PEI: Polietilenimina
PLGA: Poly(lactic-co-glycolic acid)
PNPs: Polymeric nanoparticles
PopB: *P. aeruginosa* Type III secretion system protein
ROS: Reactive oxygen species
WHO: World Health Organization
YLDs: Years of life lived with disability
